# A custom virtual reality training solution for ophthalmologic surgical clinical trials

**DOI:** 10.1186/s41077-021-00167-z

**Published:** 2021-04-16

**Authors:** Felix Heimann, Giulio Barteselli, André Brand, Andreas Dingeldey, Laszlo Godard, Hendrik Hochstetter, Michael Schneider, Alexander Rothkegel, Clemens Wagner, Joshua Horvath, Shrirang Ranade

**Affiliations:** 1 VRmagic GmbH, Mannheim, Germany; 2 Genentech, Inc., South San Francisco, CA USA

## Abstract

We present a summary of the development and clinical use of two custom designed high-fidelity virtual-reality simulator training platforms. This simulator development program began in 2016 to support the phase III clinical trial Archway (ClinicalTrials.gov identifier, NCT03677934) intended to evaluate the Port Delivery System (PDS) developed by Genentech Inc. and has also been used to support additional clinical trials. The two simulators address two specific ophthalmic surgical procedures required for the successful use of PDS and provide state-of-the-art physical simulation models and graphics. The simulators incorporate customized active haptic feedback input devices that approximate different hand pieces including a custom hand piece specifically designed for PDS implantation. We further describe the specific challenges of the procedure and the development of corresponding training strategies realized within the simulation platform.

## Introduction

The industry development of simulators in healthcare is generally focused on such surgical, procedural, or diagnostic challenges that occur with high volume and are mostly performed according to well established and possibly international standards. This combination has the best chance of producing a profitable business case which can carry the extensive costs usually associated with the development of a new high-fidelity simulation solution, especially when virtual reality (VR) technologies are involved. Consequently, the bulk of available simulator products target a rather small set of medical procedures which is particularly true for surgical simulators [1]. Custom development commissioned by industry partners from pharmaceutical and medical device industries constitute a segment of the surgical simulation market that extends the range of simulator solutions to product-specific and non-standardized procedures but remains limited to high revenue products to justify the investment.

The partnership formed between Genentech Inc. and VRmagic GmbH in the autumn 2016 deviated from this pattern in that it was formed to produce a training device for a product that was still in development and had not yet been commercialized. Genentech was about to start a phase II study for a new and innovative product, the Port Delivery System with ranibizumab (PDS), which is a new permanent and refillable eye implant, approximately the size of a grain of rice, that releases drug continuously into the vitreous of the human eye. The phase II Ladder study (ClinicalTrials.gov identifier, NCT02510794), specifically evaluated the effects of a specialized formulation of ranibizumab delivery for the treatment of neovascular (wet) age-related macular degeneration (see [2]). The initial clinical experience with the implant had indicated that the implant insertion can be performed safely as an outpatient surgical procedure. However, the implant insertion surgery involves a number of surgical steps not commonly performed by vitreo-retinal surgeons. Furthermore, the refill-exchange procedure that exchanges the implant content with fresh drug only resembles the commonly performed intravitreal injections but requires a more precise execution. To ensure patient safety and minimize the risk of surgical errors to the overall outcome of the trial, Genentech’s development team started looking for the best possible way to train the study investigators on the PDS procedures. After 2 years of intense development efforts, in the spring of 2018, Genentech and VRmagic rolled out two completely new, state-of-the-art virtual reality simulator platforms enabling its users to practice the implant insertion and the refill-exchange of the PDS, not in support of training and marketing for an existing product but as a training aid for surgeons involved in ongoing PDS clinical development.

While some training data has already been collected, including performance metrics and training meta-data, previous training sessions were focused on providing the best possible training of the trial investigators. However, protocols that would ensure an unbiased evaluation of the simulator itself were not a priority. As it is currently unclear when statistically relevant data suitable for the validation of the simulator construct might be available, this article intends to provide a concise description of the hardware and software that constitutes the simulator platform.

The list of distinct AR/VR surgery simulator systems which are consistently employed in real-life training programs is short ([3] lists 70 types of simulators over all medical fields that were developed during the last 20 years) and grows shorter if we consider only systems with active haptic feedback [4].

## The procedures

**Fig. 1:**
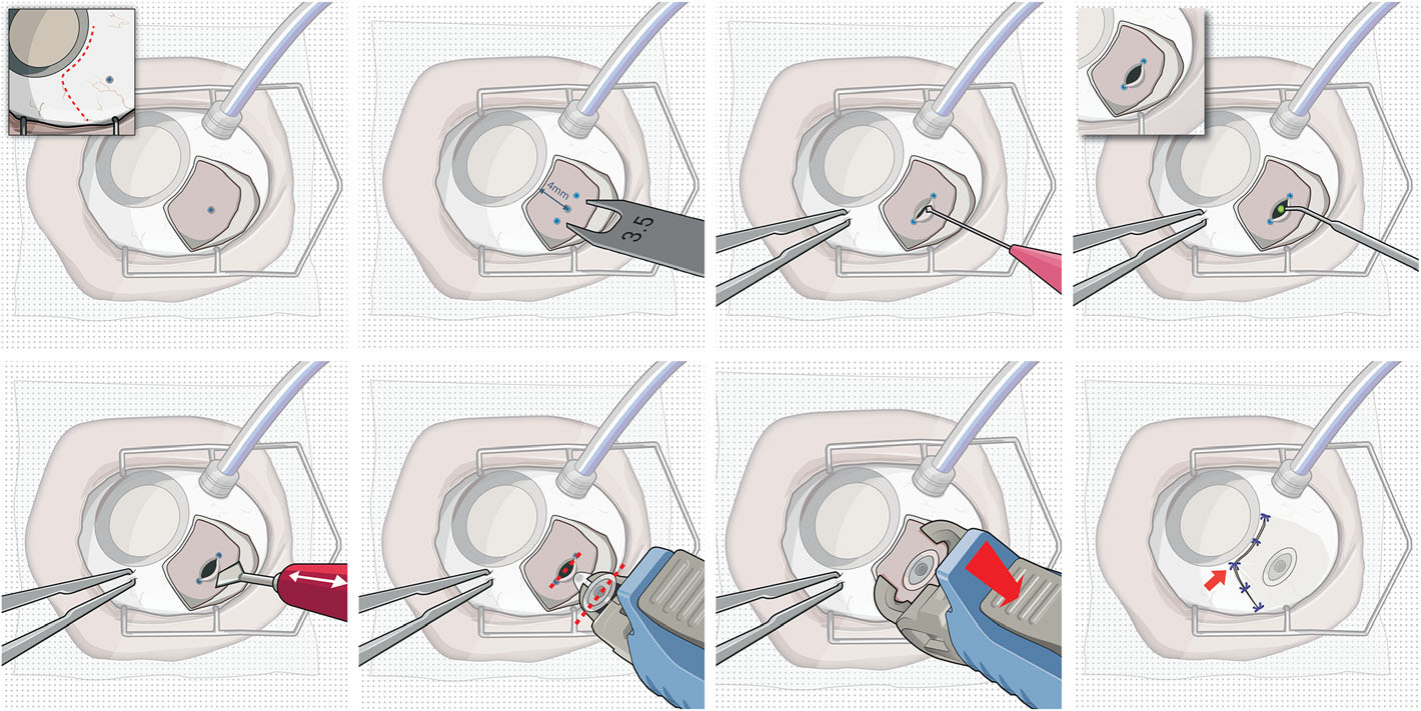
The implantation procedure requires the surgeon to perform a dissection of the conjunctiva as well as the attached Tenon’s capsule (**a**) and then mark the end points of the scleral dissection on the sclera (**b**). This is necessary as the scleral dissection (**c**) must have a length of exactly 3.5 mm with little margin for error. Afterwards, the well perfused pars plana tissue underlying the sclera needs to be coagulated (**d**) to ensure that the following pars plana incision (**e**) does not result in vitreous hemorrhage. Finally, using a special implantation tool, the Port Delivery System implant is inserted (**f**) into the globe and released from the tool (**g**). In a final step, the conjunctiva and Tenon’s capsule are closed and sutured (**h**)

The insertion procedure of the PDS implant is illustrated in Fig. 1. First, dissection of conjunctiva and Tenon’s capsule at the implant site in the superotemporal quadrant of the eye is performed. This is followed by a precise scleral dissection to expose the underlying pars plana. The vascular tissue of the pars plana tissue gets then coagulated with an ab-externo laser ablation technique [5] to mitigate vitreous hemorrhage from pars plana bleeding, a potential complication of vitreoretinal surgical procedures that generally result in vision loss [6].

**Fig. 2:**
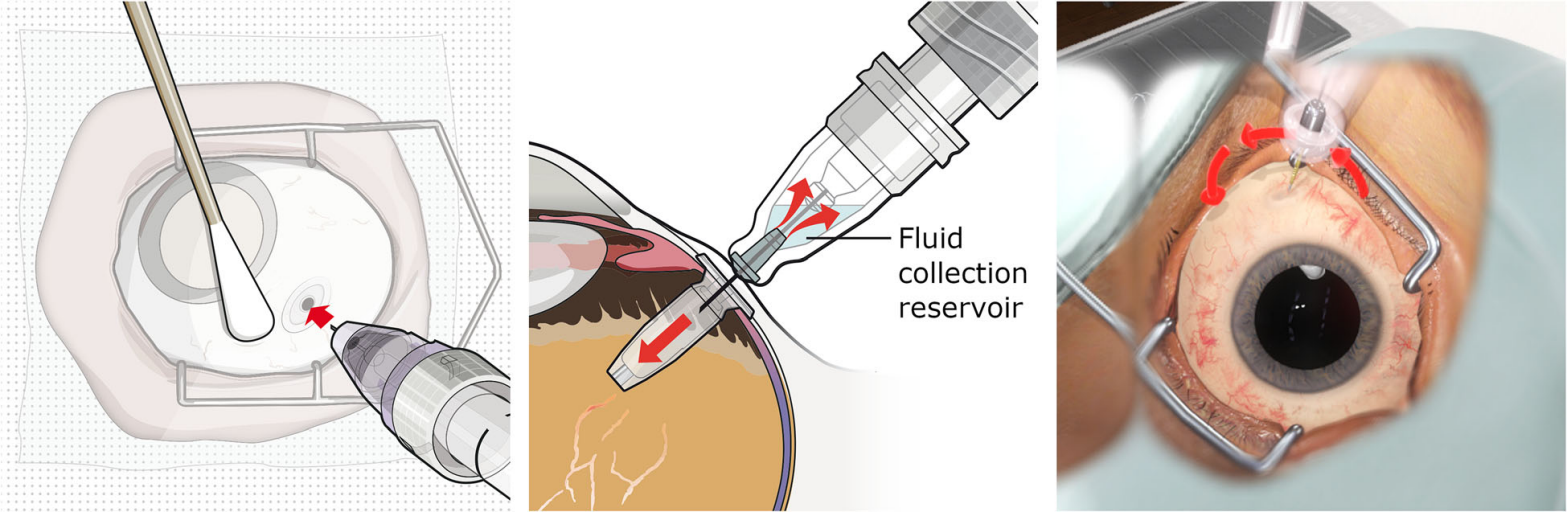
The pictures to the left and in the middle illustrate the concept of the refill-exchange procedure. A correct execution requires the full penetration of the special vented refill needle into the self-sealing septum of the PDS implant. This can only be achieved if the entry point of the needle is centralized within the septum surface and the needle is perpendicularly aligned with the implant. The picture to the right shows a simulator screenshot and illustrates the use of abstract guidance elements to help the user understand the geometric challenges of the procedure

For the refill-exchange procedure, the surgeon employs a syringe with a custom needle that includes a reflux chamber and exchanges the contents of the implant with the new drug in the syringe simultaneously. A successful refill, as illustrated in Fig. 2, requires the surgeon to insert the refill needle perpendicularly into the implant septum. With a septum diameter of about 1 mm, a complete insertion requires both a central entry point as well as orthogonal alignment of septum and needle.

## The simulator platforms

**Fig. 3:**
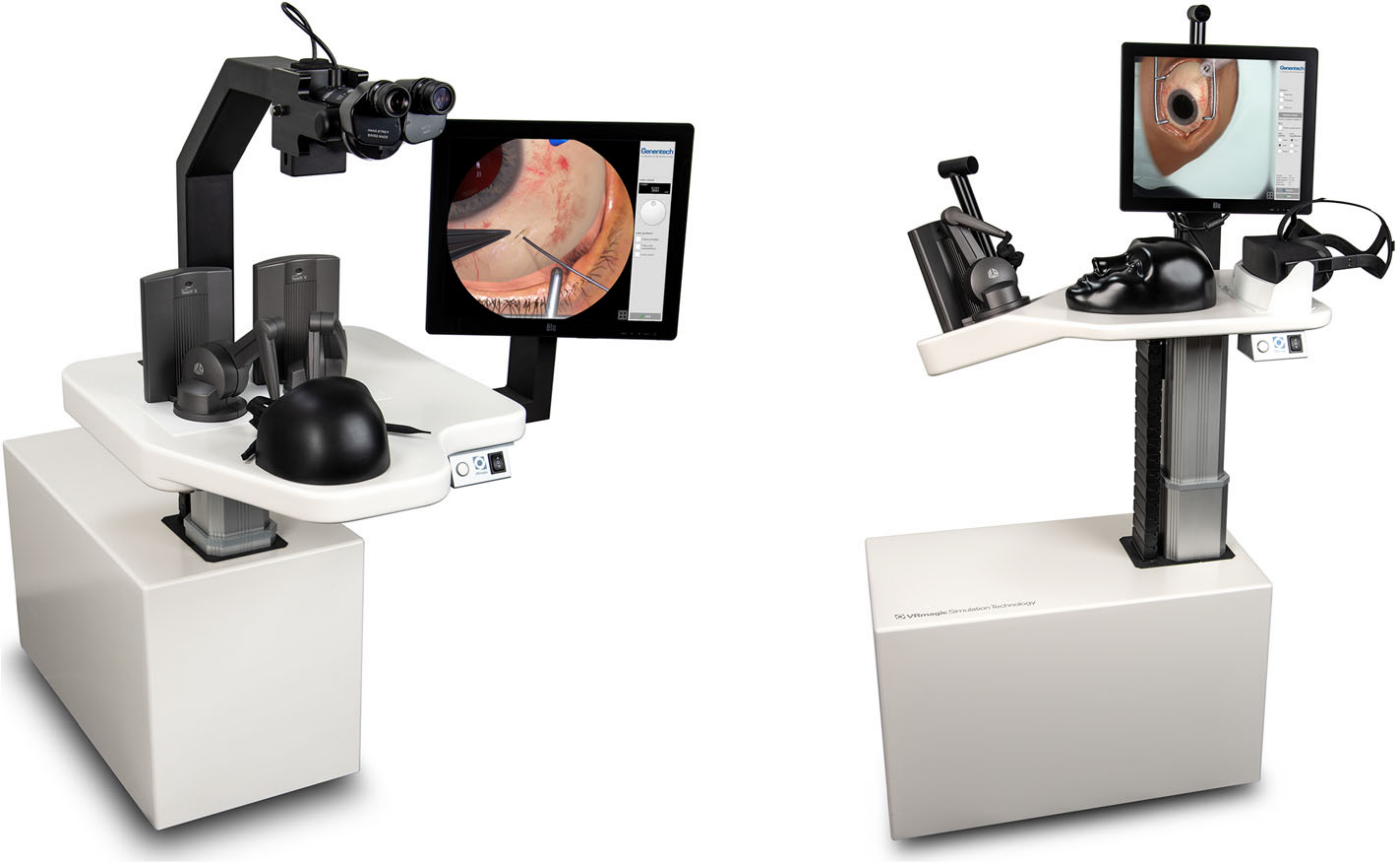
The simulator platform for the implant insertion procedure (left picture) approximates a setup in which the user sits in an OR environment and performs surgery on the virtual patient from a frontal position. Only about one quadrant of the virtual patient’s head is actually realized as a physical model, roughly modeling the part from the brow ridge to the top, which is necessary as a hand rest for the user. The simulator platform for the refill-exchange procedure (right picture) enables the user to stand next to the virtual patient who is positioned sitting in an inclined ophthalmic chair

Although their ergonomic layouts are distinctly different, the two simulator hardware platforms shown in Fig. 3 were designed with the intent of maximizing the set of shared components: both platforms are mounted on a mobile base unit that contains the computing hardware and in both cases active force feedback devices were used to capture the user input and render haptic feedback. As the development timeline did not allow the development of a custom force feedback system, both platforms employ the *TouchX* devices produced by *3D Systems*. When bought off the shelf, the device comes with a fixed stylus that is held by the user like a pen and attached to the actuated device arms via a gimbal. However, the design does not enable the user to exchange the stylus with a custom handpiece and the extent of the gimbal mechanics prohibit the usage of two devices in sufficient proximity to simulate user input during the implant insertion procedure. This specific procedure requires bimanual instrument interaction on a surgical field spanning less than one octant of a human eye. To avoid device collisions during the simulation, the gimbal and stylus parts of the device were substituted by custom components designed by the VRmagic development team with a geometric configuration optimized for ophthalmic surgery training. As the procedures require the use of different instruments that cannot be accurately represented by the same physical handpiece, the new gimbal construction was augmented to allow exchanging handpieces that can be fastened via a magnetic lock system and communicate internal degrees of freedom, e.g., the opening angle of a forceps, via spring loaded connectors. In total, four different handpieces were developed. Their shapes and internal degrees of freedom were modeled to emulate (i) forceps, (ii) a syringe, (iii) a number of simple straight instruments (e.g., scalpel or cotton tip) and (iv) a special tool designed by Genentech for the sole purpose of placing and releasing the implant.

The salient differences between the two platforms presented in Fig. 3 arise from the distinctly different contexts in which both procedures are performed. While insertion of the implant requires the surgeon to operate in an adequately prepared and staffed OR environment, the refill-exchange can be performed by a single practitioner with minimal preparation in a clinical office setting. The former requires the surgeon to sit and observe the situs through a microscope, the latter is to be performed in a standing position, slightly bent over the patient sitting in an ophthalmic chair and using only loupes for magnification. The differences in the ergonomic setup translates into different work space requirements on the haptic input devices and correspondingly different positions and orientations of these devices relative to the virtual situs which constitute the predominant constraints for the platform design.

For the implant insertion procedure, all visual feedback from the virtual scene is generated by a special stereoscopic viewing system. It was developed by VRmagic in cooperation with Haag-Streit Diagnostics and combines an actual microscope lens tube with two micro displays providing a resolution of 1080 × 1080 pixels per eye.

The development timeline prohibited any attempt of developing a custom head mounted display that would optimally simulate the loupes provided to the investigators for the refill-exchange procedure. However, by modeling the optical effects of the magnifying lenses within the graphic rendering pipeline, the requirements on the actual hardware displays could be sufficiently reduced to allow the integration of a consumer product. After weighing the benefits of the various products available by the beginning of 2018, the *Oculus Rift CV1* was selected due to the high fidelity and stability of its tracking system.

## Simulator training benefits

Today, there are various methods for simulation-based surgical training in ophthalmology including traditional wet lab training with animal models or hybrid animal/synthetic models, dry lab training on synthetic models as well as virtual reality training simulators [7]. While clinical development is still ongoing and there is not yet any data that would allow for a validation study of the simulator training, the following descriptions of some aspects of the training modules provide examples to illustrate the potential of virtual reality simulators to teach these procedures efficiently.

### Modularized learning

For the implant insertion procedure, the simulator curriculum covers only the parts with the highest impact on the clinical outcome starting with the scleral dissection and ending with the actual insertion and placement of the PDS implant. This partial procedure is split into four separate training modules comprising (i) the scleral dissection, (ii) the laser ablation of the pars plana, (iii) the pars plana incision, and (iv) the actual implant insertion.

For the refill-exchange procedure, a separation into more than one training module is not appropriate. Instead, the procedure can be performed on different virtual patients representing varying levels of difficulty essentially controlled by the visibility of the implant through the conjunctiva and the haptic resistance during insertion.

Each training module can be started directly thus enabling the user to train procedural steps out of order and focus their efforts on those tasks considered most challenging. Furthermore, the user can restart a module at any point and thus reset the scene for a new attempt instantaneously. Out of order training and immediate restarts provide a significant benefit that cannot be attained when training on animal models and constitute crucial features considering that training sessions for the trial investigators may last only 15–30 min.

### Scleral dissection

The PDS implant needs to be seated within a scleral incision of a very specific target length that corresponds to the long axis of the implant, with a minimal margin for error. Furthermore, the incision needs to be created without harming the underlying pars plana tissue. Considering that the average thickness of the scleral tissue is only ~ 0.7 mm [8], the surgeon needs to navigate his blade within a very narrow corridor while compensating for the erratic forces resulting from the toughness of the scleral tissue.

To enable a real-time simulation of this process, the simulator employs a very fine and dynamic sub-triangulation of the scleral tissue adjacent to the expanding cut. The resulting resolution is sufficient for an accurate physical simulation model and the rendering of consistent haptic feedback. A significant effort was made to obtain an accurate graphical rendering of the fibrous scleral tissue allowing the user to learn the correct interpretation of visual cues that indicate the incision depth within the scleral tissue.

**Fig. 4:**
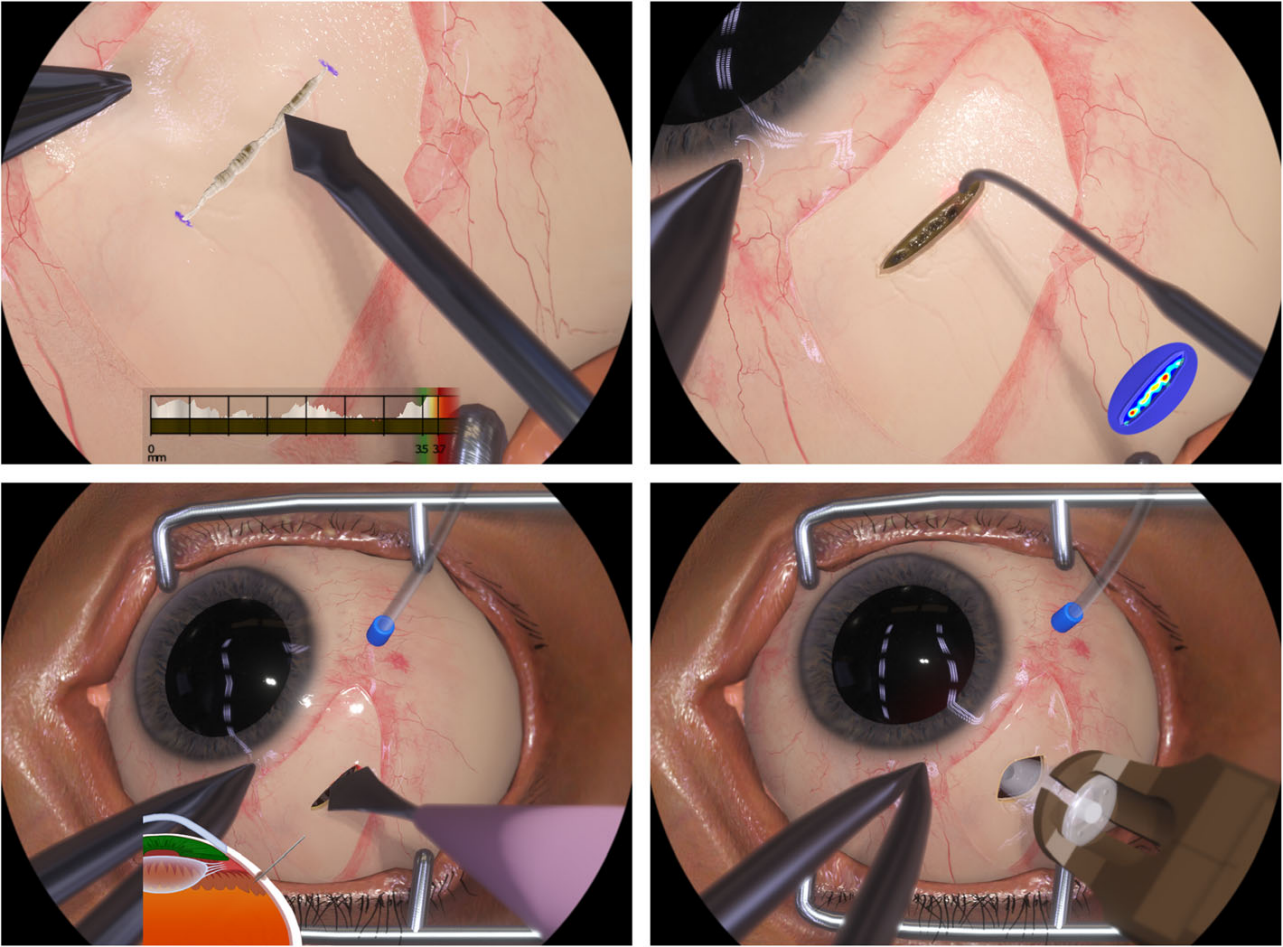
The pictures above show graphical renderings from all four training modules of the implant insertion simulator. All relevant physical effects are simulated in real time including the fluid dynamics of blood, supra choroidal fluids, and vitreous, the elastic instrument interaction with the sclera tissue, the ablation effects of the laser, and the dissection of sclera and pars plana tissue. The pictures also illustrate some of the VR guidance elements that instruct the user during training. During scleral dissection, a heads-up display (HUD) shows a profile illustrating the cut depth and length (top-left picture). During pars plana ablation, a heat map can be shown either in the periphery of the field-of-view (top-right picture) or directly over the pars plana tissue (see Fig. 5). During pars plana incision, a cross section of the supra-temporal eye quadrant illustrates the penetration depth and angle of the slit knife and its proximity to intra-ocular structures (bottom-left picture)

Compared with other conceivable training methods, a major benefit of this approach is the ability of the VR simulator to compute and report the relevant metrics in real-time as well as in a posteriori analysis. The former is provided as a heads-up display (HUD) that allows the user to directly associate subtle visual cues with objective depth information and thus improve their ability to assess the incision geometry (see the first picture in Fig. 4). The HUD further allows the user to recognize common mistakes like rounded depth profiles at the incision corners which are hard to recognize even by expert observers. A posteriori evaluation of user performance includes information about tissue treatment as well as the latitudinal and longitudinal placement of the incision.

### Laser ablation of the pars plana

The fundamental challenge of this procedure is the correct application of the laser probe which is used in a context and configuration not usually employed in vitreoretinal surgery. The ablation of the pars plana tissue needs to be applied as homogeneously as possible because the perforation of the tissue, which is achieved at the end-point of the procedure, may result in the prolapse of liquefied vitreous that will impede further treatment of yet insufficiently ablated parts of the tissue.

**Fig. 5:**
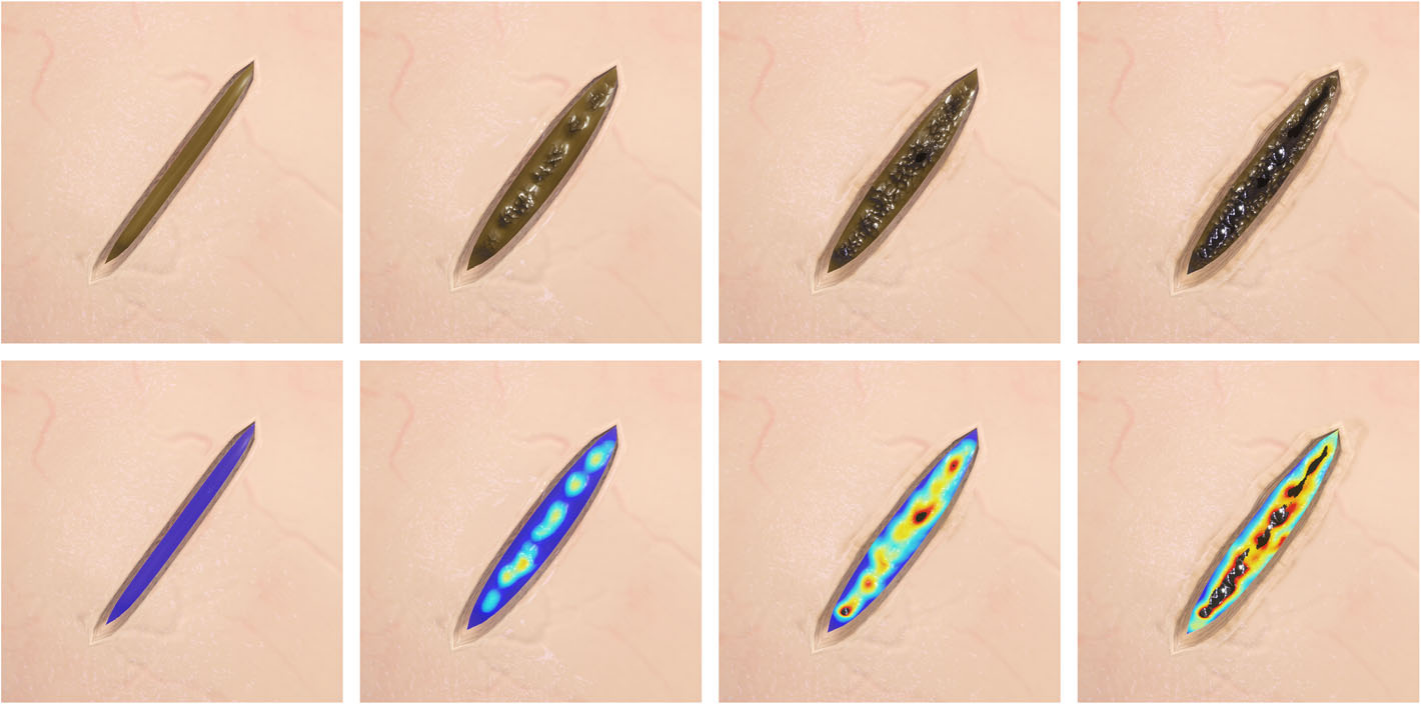
A homogeneous application of the laser to the pars plana tissue is paramount and thus the user needs to understand the visual cues that indicate the current state of ablation. The pictures above show actual renderings from the implant insertion simulator that illustrate how the laser ablation changes the appearance of the pars plana tissue. The first few laser shots provide distinct localized deformation and discoloration of the tissue. Further ablation results in the expression of a fine granular texture as well as further darkening of the tissue. Eventually, perforation of the pars plana tissue will occur and result in percolation of thick vitreous or a prolapse of liquefied vitreous. The VR simulation is sufficiently accurate to compute a heat map illustrating the degree of ablation with a high-spatial resolution and thus allows the user to train its perception of the state of ablation and ensure a homogeneous application

The VR simulator renders a highly accurate representation of the various visual indicators that reveal the current ablation state of the tissue. Using the instantaneous restart feature of the simulator, the user can experiment with different laser settings and quickly obtain an intuitive understanding of the coagulation technique. A further benefit of the VR environment is the simulator’s ability to display a heat map of the exposed pars plana tissue either as a HUD or as an overlay element (see Fig. 5).

### Refill-exchange

Potential complications during this procedure originate mostly from poor alignment of the syringe needle with the implant septum. Implementing a sufficiently accurate simulation model was a challenge due to the complex coupling of the relevant scene objects: the implant orientation is coupled to its adjacent sclera tissue and the external forces that drive this interaction result from the penetration of the septum by the syringe needle or its collision with the implant flange. As the needle is flexible, the overall system has a distinct non-linear dynamic. Hence, significant development efforts were required to render the characteristic haptic effects that guide the user during needle insertion.

Early training sessions with the simulator quickly revealed that the users had a tendency to be overconfident about their ability to visually estimate the relative orientation of implant and syringe. Exploiting VR technologies to help the user understand and correct false perceptions became a priority among the training requirements. To this effect, the simulator supports users with abstract guidance elements augmenting the virtual scene. Colored arrows and targeting markers materialize on request and guide the user into the correct position and orientation, see Fig. 2. A replay feature allows the user to observe his own performance from a different perspective and recognize the nature of the cognitive biases causing a previously experienced misperception.

## Conclusions

We report on a new and specialized simulation solution in which physicians can train on insertion and refill procedures of an eye implant for intravitreal drug delivery. This solution includes two separate and stand-alone hardware setups with distinct stereoscopic viewing systems, active force feedback input devices with customized exchangeable handpieces and multi-physics interaction models. To the authors’ knowledge, this approach constitutes a novelty and a milestone among the existing simulation solutions in Ophthalmology.

While awaiting the results of the clinical studies that are ongoing, the only indications regarding the construct validity of the simulator training is the positive feedback of the investigators and clinical experts participating in the trial and the associated simulator training sessions ([9, 10]).

To date, Genentech has employed over thirty simulator units for their training efforts.

In consideration of the high financial stakes involved in many clinical trials carried out by pharmaceutical and medical device companies, the significant investment in high-fidelity training devices to support surgical training and thus enable commercialization of new products may be rapidly amortized if there is a measurable improvement of the clinical outcome. Nevertheless, the authors are not aware of any recent trials that were accompanied by similar developments. This publication is at least partially intended to draw attention to this particular use case: the support of clinical trials with VR-based high-fidelity simulators has enormous potential to extend their range of applications to new medical fields and operations for which such a development would otherwise be cost prohibitive.

## Data Availability

N/A
